# Comparison of Recreational Fish Consumption Advisories Across the USA

**DOI:** 10.1007/s40572-021-00312-w

**Published:** 2021-05-01

**Authors:** Brittany M. Cleary, Megan E. Romano, Celia Y. Chen, Wendy Heiger-Bernays, Kathryn A. Crawford

**Affiliations:** 1 Dartmouth College, Hanover, NH USA; 2 Department of Epidemiology, Geisel School of Medicine at Dartmouth, Lebanon, NH USA; 3 Department of Biological Sciences, Dartmouth College, Hanover, NH USA; 4 Department of Environmental Health, Boston University School of Public Health, Boston, MA USA; 5 Program in Environmental Studies, Middlebury College, Middlebury, VT USA

**Keywords:** Fish consumption advisory, Methylmercury (MeHg), Polychlorinated biphenyls (PCBs), Self-caught fish, Target tissue concentration

## Abstract

**Purpose of Review:**

Our comparative analysis sought to understand the factors which drive differences in fish consumption advisories across the USA — including exposure scenarios (acute and chronic health risk, non-cancer and cancer health endpoints), toxicity values (reference dose, cancer slope factor, acute tolerance level), and meal size and bodyweight assumptions.

**Recent Findings:**

Fish consumption provides essential nutrients but also results in exposure to contaminants such as PCBs and methylmercury. To protect consumers from the risks of fish contaminants, fish consumption advisories are established, most often by state jurisdictions, to estimate the amount of a certain fish species a person could consume throughout their lifetime without harm. However, inconsistencies in advisories across the USA confuse consumers and undermine the public health goals of fish advisory programs. To date, no rigorous comparison of state and national fish consumption advisories has been reported.

**Summary:**

Our work identifies discrepancies in key assumptions used to derive risk-based advisories between US states, reflecting differences in the interpretation of toxicity science. We also address the implications for these differences by reviewing advisories issued by contiguous states bordering two waterbodies: Lake Michigan and the Lower Mississippi River. Our findings highlight the importance of regional collaboration when issuing advisories, so that consumers of self-caught fish are equipped with clear knowledge to make decisions to protect their health.

**Supplementary Information:**

The online version contains supplementary material available at 10.1007/s40572-021-00312-w.

## Introduction

Fish consumption presents a critical dilemma to consumers, communities, and healthcare professionals. Aquatic organisms are the largest dietary source of ω-3 polyunsaturated fatty acids, which improve heart health and cognition; reduce risk of lung, prostate, and colorectal cancer; and decrease risk of allergies in children [1–3]. Yet, fish consumption may also expose people to harmful environmental contaminants that bioaccumulate in fish tissue [4]. Methylmercury is a widespread and potent toxicant synthesized by microbial transformation of inorganic mercury, which enters aquatic systems from adjacent watersheds and atmospheric deposition [5]. In the USA, people are primarily exposed to methylmercury through seafood consumption [6]. Methylmercury is associated with numerous adverse health effects, including impaired neurological, reproductive, and immunological health [7–10]. Notably, perinatal methylmercury exposure, including at low concentrations, is associated with hindered cognitive and psychomotor performance in children [11, 12]. The impact of methylmercury exposure on intelligence quotient (IQ) poses a significant financial burden to society, estimated to be more than $4.7 billion annually in the USA alone [13, 14]. Polychlorinated biphenyls (PCBs) are a group of compounds which mainly contaminate waterbodies near industrial areas [5]. PCBs, like methylmercury, are associated with a wide range of adverse health effects, including cancer and impaired reproductive, immunological, endocrine, hepatic, cardiometabolic, and neurological health [15–20]. PCB exposure is ubiquitous among the general US population, with diet regarded as the primary source [19, 21–23]. Like methylmercury, the burden of disease posed by PCBs is also significant. Many researchers have proposed models for balancing the positive and negative health effects of fish consumption, but a robust public health program must be implemented to educate and engage consumers about safe fish consumption in a way that promotes health benefits while minimizing potential risks [24–27].

In the USA, public health and/or environmental protection agencies of individual states issue fish consumption advisories (“advisories”) to help consumers of self-caught fish and shellfish (referred to as “fish” throughout) make informed decisions regarding health risks posed by contaminants in fish. Generally, advisories provide guidance about the maximum amount of fish that a person could safely consume from a waterbody, given concentrations of contaminants measured in locally caught fish and associated health risks [28]. Recommendations may range from no advisory — if contaminant concentrations are low enough for safe consumption at any quantity — to zero consumption, if contaminant concentrations are too high for any level of safe consumption. Often, less-protective advisories are issued for the general population (GP; e.g., adult males and women beyond childbearing age), whereas separate, more protective advisories are issued for sensitive populations (SP; e.g., women of childbearing age and young children) because the health risks posed by exposure to contaminants have the potential to be greater among these individuals [29•]. Other particularly vulnerable subpopulations are high-frequency consumers of self-caught fish, a group comprised disproportionately of minority and low socioeconomic status individuals who may be at elevated risk of exposure to contaminants in fish [30, 31]. Further, geographic region and cultural dietary norms may also influence an individual’s motive for eating self-caught fish [32].

Advisories are usually issued on a species-by-species basis through a risk-based approach in which interdisciplinary science about exposures and outcomes is integrated and applied to public health practice*.* The goal of the risk assessment process is to estimate the amount of a certain fish a person could consume throughout their lifetime without harm [33]. Findings are then used to inform risk management decisions and set policy, including fish consumption limits. The risk assessment process dates back to the 1980s and can be employed to evaluate risks posed by an array of potential hazards [34]. The process by which a risk-based consumption advisory is developed requires the incorporation of four key variables: (1) toxicity values, either (a) the reference dose, a quantitative estimate of the daily oral exposure to a specific contaminant below which adverse effects are not expected to occur or are extremely unlikely in an exposed population [35], or (b) the cancer slope factor, a quantitative estimate of the increase in cancer risk associated with oral exposure to a contaminant [35]; (2) policy-based acceptable risk levels for (a) chemicals with non-cancer effects where the hazard quotient is the ratio of the estimated exposure to a contaminant and the level at which adverse health effects are not expected (the reference dose) and (b) chemicals with documented cancer outcomes where the target cancer risk level is the estimated lifetime cancer risk from ingesting a carcinogenic contaminant; (3) bodyweight, representative of the population for which acceptable contaminant concentrations are derived; and (4) consumption rate, representing the average daily fish intake corresponding to a specific number of meals of fish consumed over a defined time period [36, 37]. The resulting concentration of a contaminant is then compared to actual measurements of the contaminant in fish collected from a particular waterbody to evaluate whether that fish is safe for a person to eat. Assumptions about the science of contaminant toxicity, acceptable human health risk, and characteristics of an average person are embedded within each of these variables. In 2009, the National Research Council (NRC) proposed improvements to risk assessment to ensure that the assessments make use of the best available science, are technically accurate, and are most relevant for decision-making [38]. To date, these recommendations have not been widely adopted and are not incorporated into fish consumption advisories. As a result, our current fish consumption advisories may not reflect the current state of the science nor be adequately protective of the populations they seek to protect.

Fish consumption advisories are calculated separately for each contaminant present in fish tissue. The contaminant which yields the most health-protective estimate of how much fish an individual can safely consume dictates the advisory published for a given waterbody [39]. Advisories for methylmercury are typically applied on a state-wide basis since atmospheric transport of mercury leads to wide-spread geographic distribution of methylmercury [5]. Highly polluted waterbodies may receive site-specific methylmercury advisories. By contrast, PCBs are typically associated with hazardous waste discharges and are less mobile in the environment [5], so are most often subjects of site-specific rather than state-wide advisories.

Multiple regulatory and cooperative organizations publish guidance on how to employ risk assessment techniques to derive advisories based on chronic non-cancer and cancer health endpoints. Regulatory organizations include the US Environmental Protection Agency (EPA) and the US Agency for Toxic Substances and Disease Registry (ATSDR); cooperative organizations include the Great Lakes Consortium (GLC), a collaboration of fish advisory managers from states surrounding the Great Lakes [28, 40–43]. The US Food and Drug Administration (FDA) publishes broad fish tissue contaminant guidance protective of acute, short-term health [44]. States may also choose to develop their own fish consumption advisory guidance. Nuanced differences exist between these approaches and can lead to discrepancies between advisories across jurisdictional boundaries. This presents a challenge for consumers of self-caught fish, especially those that fish in both their state of residence and other states, a group which represents about 14 percent of people who fish [45]. The environmental health and risk assessment communities have called for uniformity in advisory assumptions and dissemination since the 1990s [29•, 46•, 47•, 48–49]. Efforts to streamline public health messaging have clarified some confusion among consumers of self-caught fish [50–54], yet these groups may still exceed advisory guidelines or avoid consuming any fish because they are confused by public health messaging [55–58]. Ensuring that advisory messages are consistent across jurisdictional boundaries, especially within a single waterbody, is one avenue to increase the credibility of advisories among consumers of self-caught fish and thus their efficacy for protecting public health.

To better understand differences in advisories across jurisdictional boundaries, we examined (1) risk assessment assumptions, represented by values of risk assessment variables, used to derive advisories for methylmercury and PCBs across US states; (2) the target fish tissue concentrations of methylmercury and PCBs that warrant an advisory of one meal per month (meal/month) or zero consumption across states; and (3) advisories for common sport fish in two waterbodies bordered by multiple states, Lake Michigan and the Lower Mississippi River. Our work summarizes how risk assessment assumptions can drive variation in advisories and highlights how standardized approaches improve public health messaging about safe fish consumption. Despite the complex health risks associated with their consumption, fish are important sources of protein and nutrients, so the need to engage communities and stakeholders with clear and consistent advisories remains critical.

## Methods

### Collection and Review of US State Advisory Guidance Documents

In order to evaluate the assumptions that each state makes while establishing advisories for the consumption of self-caught fish, we used a standardized, multi-step approach to obtain state technical guidance documents, which consisted of (1) review of a state’s advisory website; (2) direct communication with a state’s risk assessment personnel; and (3) an Internet search with pre-specified search criteria. State fish consumption advisory websites and personnel contact information were available via the EPA *State, Territory and Tribe Fish Advisory Contacts* list [50]. All document searches and contact with state personnel occurred during 2019. Through this approach, we obtained and reviewed technical guidance documents for 46 of 50 US states. The remaining four states (Montana, North Carolina, South Dakota, Texas) did not have technical guidance documents available on their advisory website and did not respond to our requests for information.

### Comparison of Risk Assessment Assumptions Across US States

We focused our comparison on two environmental contaminants, methylmercury and PCBs, which collectively represent the majority of fish consumption advisories issued in the USA [46•]. We reviewed each state’s technical guidance document and abstracted information about the following four risk assessment variables: toxicity values (reference dose, cancer slope factor, acute tolerance level), risk levels (cancer risk level, target hazard quotient), bodyweight, and meal size. The value of each variable represents assumptions about the science of contaminant toxicity, acceptable human health risk, and characteristics of an average person. We recorded separate values for the GP and SP (women of childbearing age, young children under 6 years of age, and the overall SP) when distinguished in state guidance documents. While states may also consider other variables — including exposure timing variables, absorption factors, and cooking reduction factors — we focused our analysis on these four variables because of their importance in calculating human health risk from consuming self-caught fish. These risk assessment variables are used to derive a target tissue concentration for chronic non-cancer and chronic cancer health endpoints, which represents the theoretical concentration of methylmercury or PCBs in fish tissue that would trigger an advisory:

1 $$ C\left(\frac{mg}{kg}\right)=\frac{RfD\left(\frac{mg}{kg\times day}\right)\times BW(kg)\times THQ}{CR\left(\frac{kg}{day}\right)} $$

for chronic non-cancer health endpoints

and

2 $$ C\left(\frac{mg}{kg}\right)=\frac{RL\times BW(kg)}{CSF\left(\frac{kg\times day}{mg}\right)\times CR\left(\frac{kg}{day}\right)} $$

for chronic cancer health endpoints [36, 37].

where C = target tissue concentration (mg/kg), RfD = reference dose (mg/kg-day), BW = bodyweight (kg), THQ = target hazard quotient (unitless), CR = consumption rate (kg/day), RL = target cancer risk level (unitless), and CSF = cancer slope factor ((mg/kg-day)^−1^). The target tissue concentration may be calculated for multiple consumption rates and compared against measured fish tissue contaminant concentrations to determine whether an advisory is warranted, and if so, at what consumption rate. The consumption rate may be replaced by the equivalent quantity:

3 $$ CR\ \left(\frac{kg}{day}\right)=\frac{MS\left(\frac{kg}{meal}\right)\times MF\left(\frac{ meal s}{month}\right)}{AT\left(\frac{ day s}{month}\right)} $$

where MS = meal size (kg/meal), MF = meal frequency (meals/month), and AT = averaging time (days/month). Averaging time is a unit conversion factor typically set to 30.44 days/month [28]. Equations 1 and 2 produce deterministic risk estimates, providing a single estimate of risk without indication of uncertainty or variability [59].

We calculated summary statistics for each variable to assess differences between US states. States that did not report a particular variable were omitted from the corresponding summary statistic. We also abstracted variables and assumptions from the EPA, GLC, ATSDR, and FDA technical guidance documents [28, 40–42, 44]. We conducted a sensitivity analysis to determine the influence of each variable on Equations 1 and 2. To do so, we used the minimum and maximum values in our dataset for each variable to calculate the target tissue concentrations which would hypothetically trigger an advisory of one meal/month, while holding all other variables at their median value. Relative contribution of each variable was determined as the largest difference between least and most protective simulated target tissue concentrations. We also evaluated the association between target tissue concentrations and guidance document publication year by linear regression.

### Comparison of Target Tissue Concentrations Across US States

We compared the target tissue concentrations of methylmercury and PCBs which warrant advisories in two meal frequency categories: one meal/month and zero consumption. These meal frequency categories were selected to be representative of a state’s overall advisory program. We directly abstracted target tissue concentrations from state technical guidance documents, when available: 25 states (methylmercury, one meal/month), 31 states (methylmercury, zero consumption), 22 states (PCBs, one meal/month), and 27 states (PCBs, zero consumption) (Supplemental Table S1). When not explicitly provided, we used risk assessment assumptions outlined in a state’s technical guidance document to calculate target tissue concentrations. Calculations were performed for 10 states, 7 for both methylmercury and PCBs (Delaware, Georgia, Idaho, Louisiana, Maryland, Nebraska, Oregon) and 3 for methylmercury only (New Hampshire, Utah, Washington) (Supplemental Table S1). Methylmercury calculations were not performed for four states (Florida, Rhode Island, Massachusetts, Vermont) and PCBs calculations were not performed for six states (Florida, Rhode Island, Massachusetts, Vermont, Arkansas, New Hampshire) due to lack of available information in guidance documents. Unless otherwise specified, we used EPA meal frequency rounding protocols and conversion factors in calculations [28].

### Regional Differences in Advisories

To evaluate how differences in advisory assumptions affect guidance presented to fish consumers, we considered advisories issued by states bordering two waterbodies: Lake Michigan (Illinois, Michigan, Minnesota, and Wisconsin) and the Lower Mississippi River (Arkansas, Kentucky, Missouri, and Tennessee). These waterbodies are large, popular for fishing, and bordered by four contiguous states in close proximity. We examined the differences in advisories between states for common sport fish species in Lake Michigan (brown trout, Chinook salmon, coho salmon, lake trout, rainbow trout, and yellow perch) [60–67] and the Lower Mississippi River (channel catfish, flathead catfish, blue catfish, common carp, black crappie, white crappie, bluegill, freshwater drum, largemouth bass, white bass, and striped bass) [68–73]. We also compare advisories issued by each state for a 20-inch lake trout in Lake Michigan and a 20-inch common carp in the Lower Mississippi River to demonstrate how advisories vary across jurisdictional boundaries.

## Results

### Risk Assessment Variables Differ Across US States

Of the 46 states for which we obtained and reviewed advisory technical guidance documents, 45 (96%) developed methylmercury advisories and 40 (84%) developed PCB advisories (Table 1). Most recent publication year of methylmercury and PCBs technical guidance documents range from 1992 to 2019 (Table 1; Supplemental Fig. S1a and S1b). Toxicity values (reference doses, cancer slope factors, acute tolerance levels) and cancer risk levels employed in risk assessment calculations differ across the USA and impact resulting advisories. Among the 45 states that assess methylmercury, 39 (82%) consider the chronic non-cancer health risk when developing advisories, with reference doses ranging from 7 × 10^−5^ to 5.6 × 10^−4^ mg/kg-day; three (7%) consider acute health risk, with acute tolerance levels ranging from 1.0 to 1.5 mg/kg; and three (7%) do not specify their risk scenario (Table 1; Supplemental Fig. S2a and S3a). Of the 40 states that consider PCBs, 13 (33%) consider chronic non-cancer health risk, with reference doses ranging from 2 × 10^−5^ to 5 × 10^−5^ mg/kg-day; and 23 (58%) use a chronic cancer risk-based approach, with cancer slope factors ranging from 2 to 7.7 kg-day/mg and target cancer risk levels ranging from 10^−6^ to 10^−4^ (Table 1; Supplemental Figs. S2b, S3b, S4a, S4b). Two (5%) consider an acute risk scenario based on an acute tolerance level of 2 mg/kg, and 3 (7%) do not specify their risk scenario (Table 1; Supplemental Fig. S2b). Across all states which considered the chronic non-cancer health impacts of PCBs and/or methylmercury, the target hazard quotient was set to 1. This reflects a science policy decision that contaminant exposure at a dose equivalent to the reference dose represents an acceptable level of human health risk.

**Table 1:** Summary of fish consumption advisory data collected for 46 states

Advisory characteristic	*n* states (%)	Range	Median
Contaminants advisory guidance	46 (100)	–	–
Year of guidance document publication	42 (91)	1992–2019	2012
BW	39 (85)	–	–
GP	39 (100)	60–80 kg	70 kg
Young children	11 (28)	11.6–35 kg	15 kg
WCBA	15 (38)	60–70 kg	62 kg
MS	39 (85)	–	–
GP and WCBA	39 (100)	0.113–0.283 kg	0.227 kg
Children	9 (23)	0.039–0.116 kg	0.085 kg
Methylmercury advisory guidance	45 (96)	–	–
Chronic non-cancer risk	39 (82)	–	–
RfD	37 (95)	7 × 10^−5^–5.6 × 10^−4^ mg/kg-day	1 × 10^−4^ mg/kg-day
THQ	39 (100)	1	1
Acute risk	3 (7)	–	–
TL	3 (100)	1.0–1.5 mg/kg	1 mg/kg
Unspecified risk scenario	3 (7)	–	–
PCBs advisory guidance	40 (84)	–	–
Chronic non-cancer risk	13 (33)	–	–
RfD	12 (92)	2 × 10^−5^ – 5 × 10^−5^ mg/kg-day	5 × 10^−5^ mg/kg-day
THQ	13 (100)	1	1
Chronic cancer risk	23 (58)	–	–
CSF	19 (83)	2–7.7 (mg/kg-day)^−1^	2 (mg/kg-day)^−1^
RL	22 (96)	10^−6^–10^−4^	10^-5^
Acute risk	2 (5)	–	–
TL	2 (100)	2 mg/kg	2 mg/kg
Unspecified risk scenario	3 (7)	–	–

Advisory characteristics were abstracted from individual state technical guidance documents, when available. Note: some states may employ multiple simultaneous approaches or do not apply any approach at all, such that not all categories below will add to 100%. PCB advisory guidance represents 40 states but 41 data points — New Jersey GP calculations consider chronic cancer risk, and SP calculations consider chronic non-cancer risk. *BW* bodyweight (kg), *GP* general population, *SP* sensitive populations, *WCBA* women of childbearing age, *MS* meal size (kg/meal), *RfD* reference dose (mg/kg-day), *THQ* target hazard quotient (unitless), *ATL* acute tolerance level (mg/kg), *CSF* cancer slope factor (kg-day/mg), *RL* target cancer risk level (unitless)

Additional variables that differ across the USA are bodyweight and meal size (Table 1). Of 39 states which report assumptions about average bodyweight, all reported bodyweight values for the GP, ranging from 60 to 80 kg; 11 (28%) reported bodyweight values for young children, ranging from 11.6 to 35 kg; and 15 (38%) reported bodyweight values for women of childbearing age, ranging from 60 to 70 kg (Table 1; Supplemental Fig. S5a, S5b, and S5c). Of 39 states which report meal size assumptions, all reported meal size values for the GP and women of childbearing age, ranging from 0.113 to 0.283 kg, and nine (23%) reported meal size values for young children, ranging from 0.039 to 0.116 kg (Table 1; Supplemental Fig. S6a and S6b).

Based on our sensitivity analysis for methylmercury advisories, the reference dose has the greatest influence on the predicted target tissue concentration. By contrast, the cancer risk level has the largest impact on the predicted target tissue concentration for cancer-based PCB assessments. Cancer health endpoints for PCBs were more health-protective than non-cancer health endpoints and thus are more likely to drive advisories for states which evaluate both scenarios (Supplemental Fig. S7a, S7b, and S7c).

### Methylmercury and PCB Target Tissue Concentrations Differ Across US States

**Fig. 1:**
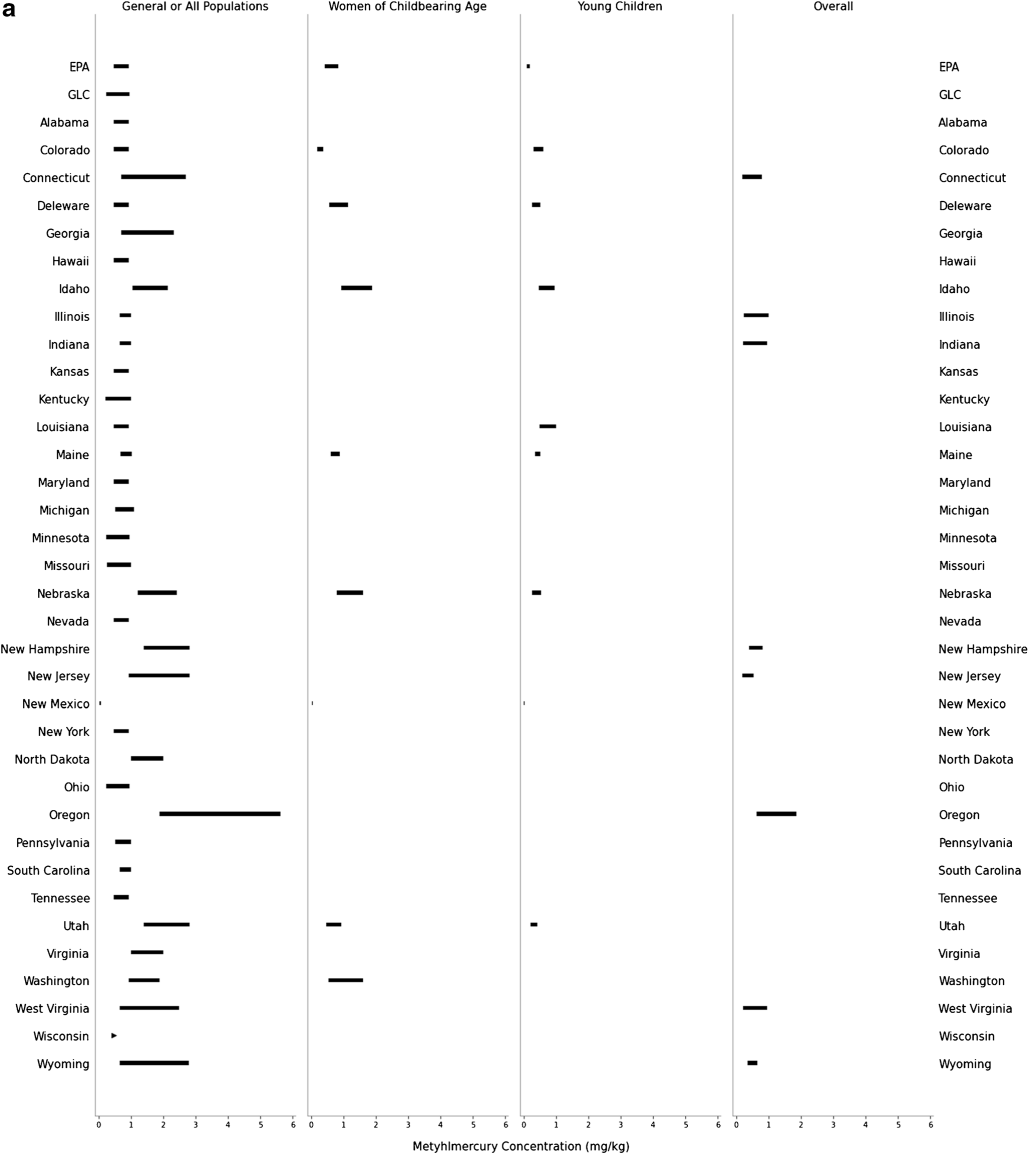

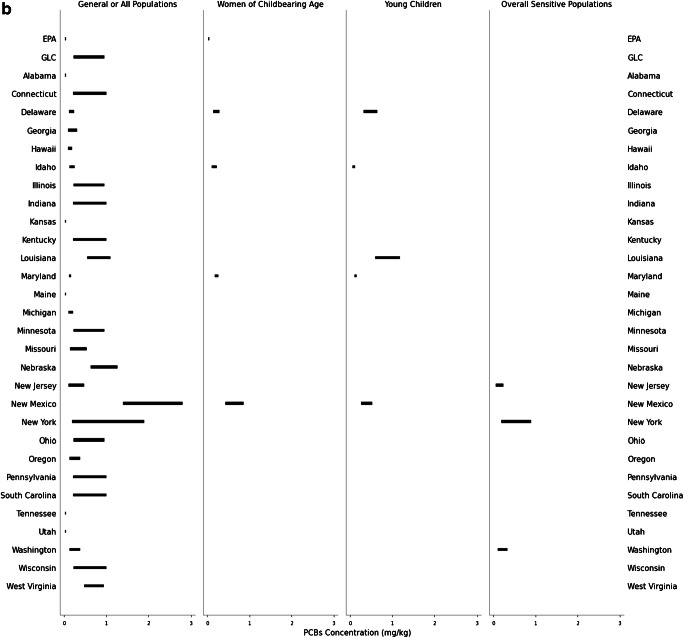
**a**–**b** Target tissue concentration range corresponding to a one meal/month fish consumption advisory. **a** Methylmercury target tissue concentration range. GP: *n* = 35 states. Women of childbearing age: *n* = 7 states. Young children: *n* = 8 states. Overall SP: *n* = 8 states. Note: Wisconsin does not have an upper-bound limit for target tissue concentrations triggering a one meal/month advisory for the GP (most protective value indicated by triangle). **b** PCB target tissue concentration range. GP: *n* = 29 states. Women of childbearing age: *n* = 4 states. Young children: *n* = 5 states. Overall SP: *n* = 3 states. See Supplemental Table S1 for detailed information about abstracted and calculated values

**Fig. 2:**
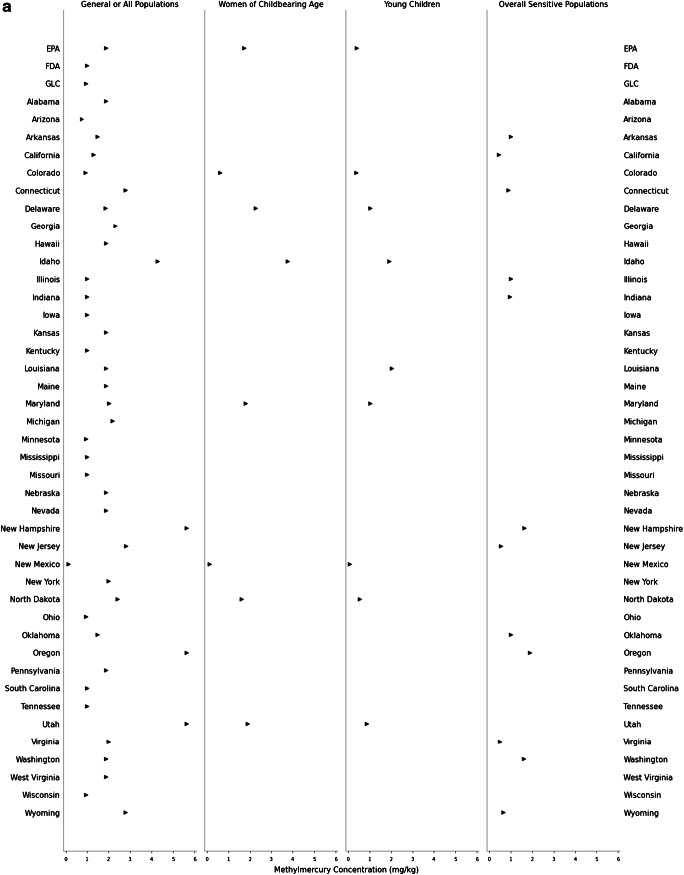

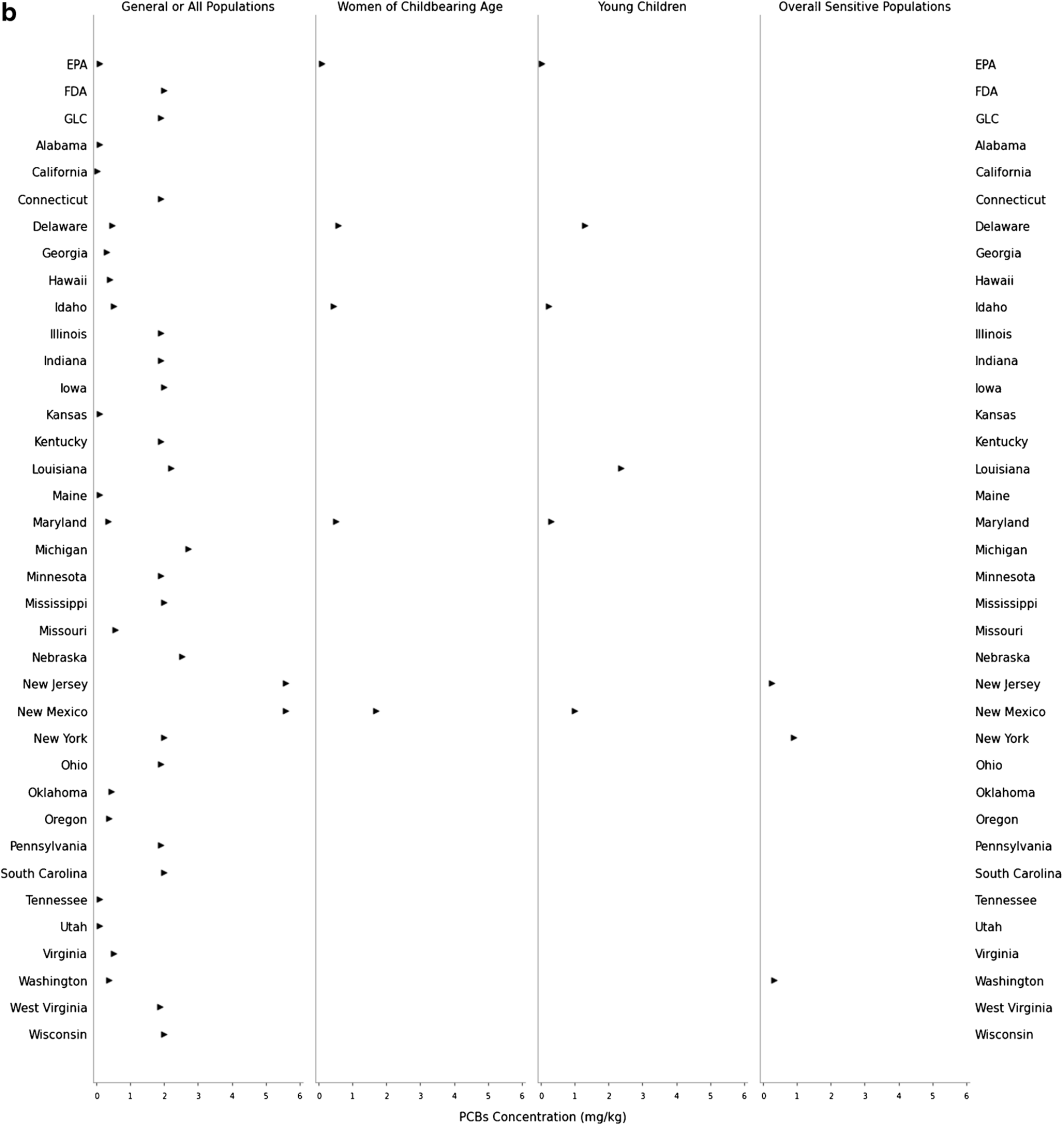
**a**–**b** Most protective target tissue concentration corresponding to a zero consumption fish consumption advisory. **a** Methylmercury. GP: *n* = 41 states. Women of childbearing age: *n* = 7 states. Young children: *n* = 8 states. Overall SP: *n* = 12 states. **b** PCBs. GP: *n* = 34 states. Women of childbearing age: *n* = 4 states. Young children: *n* = 5 states. Overall SP: *n* = 3 states. See Table S1 for detailed information about abstracted and calculated values

Consistent with discrepancies between state advisory assumptions, the target tissue concentrations warranting one meal/month or zero consumption advisories for methylmercury and PCBs also differ across the USA. The lowest and highest target tissue concentrations reflect the most and least protective advisories, respectively. The least protective concentration of methylmercury for a one meal/month advisory ranges from 0.067 to 5.6 mg/kg for the GP (*n* = 35 states); 0.061 to 1.9 mg/kg for women of childbearing age (*n* = 7 states); 0.037 to 1.0 mg/kg for young children (*n* = 8 states); and 0.54 to 1.9 mg/kg for the overall SP (*n* = 8 states; Fig. 1a). For PCBs, the least protective concentration for a one meal/month advisory ranges from 0.047 to 2.8 mg/kg for the GP (*n* = 29 states); 0.043 to 0.86 mg/kg for women of childbearing age (*n* = 4 states); 0.010 to 1.2 mg/kg for young children (*n* = 5 states); and 0.24 to 0.90 mg/kg for the overall SP (*n* = 3 states; Fig. 1b). The fish tissue methylmercury concentration warranting a zero consumption advisory ranges from 0.13 to 5.6 mg/kg for the GP (*n* = 41 states); 0.12 to 3.8 mg/kg for women of childbearing age (*n* = 7 states); 0.074 to 2.0 mg/kg for young children (*n* = 8 states); and 0.44 to 1.9 mg/kg for the overall SP (*n* = 12 states; Fig. 2a). Finally, the PCB concentration for a zero consumption advisory ranged from 0.012 to 5.6 mg/kg for GP (*n* = 34 states); 0.09 to 1.7 mg/kg for women of childbearing age (*n* = 4 states); 0.02 to 2.4 mg/kg for young children (*n* = 5 states); and 0.24 to 0.90 mg/kg for the overall SP (*n* = 3 states; Fig. 2b). The year of technical guidance document publication did not predict target tissue concentrations.

### Regional Differences in Advisories

**Fig. 3:**
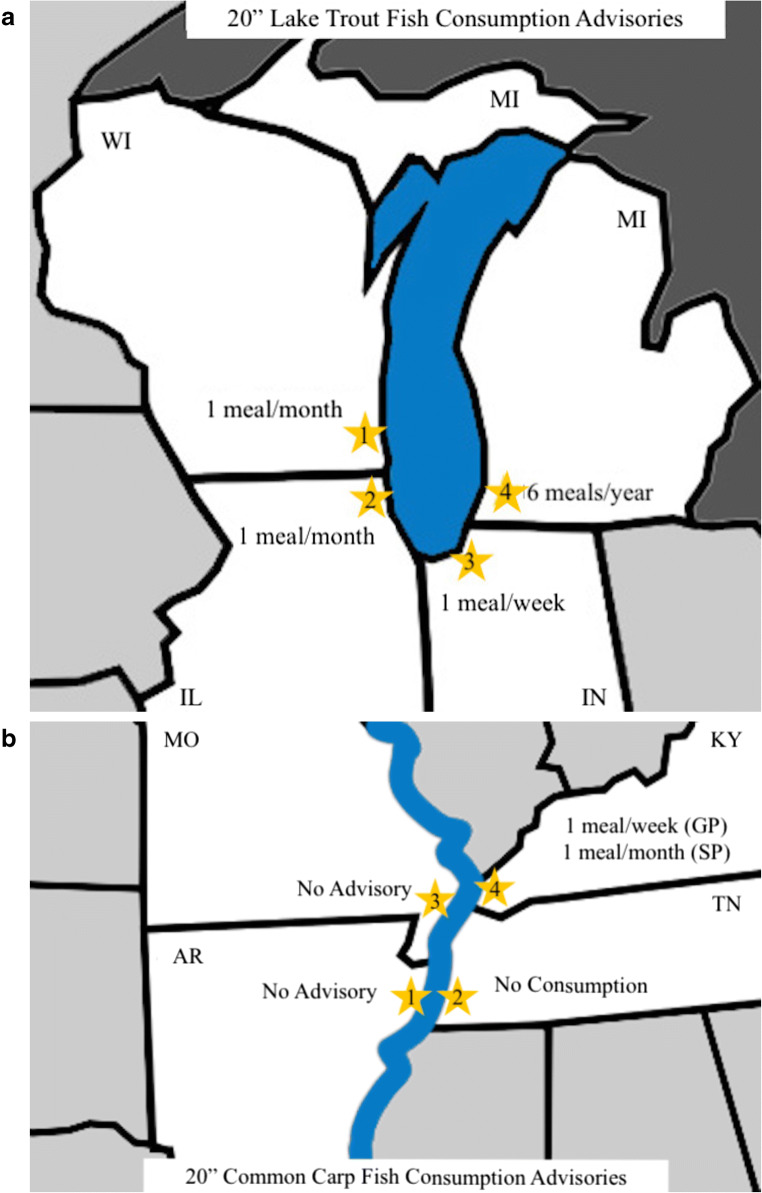
**a–b** Differences in advisories issued by states bordering a single waterbody. **a** Map of Lake Michigan showing (1) Wisconsin, (2) Michigan, (3) Indiana, and (4) Illinois. Text within each map indicates the fish consumption advisory issued for a 20” lake trout. **b** Map of the Mississippi River showing (1) Arkansas, (2) Tennessee, (3) Missouri, and (4) Kentucky. Text within each map indicates the fish consumption advisory issued for a 20” common carp

We identified variation between advisories for six common sport fish species in the four states that border Lake Michigan (Illinois, Indiana, Michigan, Wisconsin; Table 2) and 11 common sport fish species in the four states that border the Lower Mississippi River (Arkansas, Tennessee, Missouri, Kentucky; Table 3). However, the degree of variability was different between the waterbodies. Notably, if a self-caught fish consumer was to catch a 20” lake trout in Lake Michigan, they would receive slightly different messages depending on the state in which they fish, despite being in close proximity: one meal/month advisory in Wisconsin or Illinois; one meal/week in Indiana; or six meals/year in Michigan (Fig. 3a). By contrast, Arkansas, Tennessee, Missouri, and Kentucky have greater disparities in advisories for the Lower Mississippi River. A self-caught fish consumer in Arkansas who catches a 20” common carp from the Mississippi River would not find an advisory for their fish, whereas an individual a few miles due east in Tennessee would find a zero consumption advisory (Fig. 3b). If someone in Missouri were to catch a 20” common carp from the Mississippi River, they would not be advised to limit consumption, but a self-caught fish consumer nearby, in Kentucky, would be informed to limit their consumption of that type of fish to one meal/week (GP) or one meal/month (SP).

**Table 2:** Fish consumption advisories for six common game fish in Lake Michigan

	Illinois	Indiana	Michigan	Wisconsin
Brown trout	1 meal/month	1 meal/month	Limited*	1 meal/month
Chinook salmon	1 meal/month	1 meal/month	6 meals/year	1 meal/month
Coho salmon	1 meal/week (< 24")	1 meal/week (≤ 24")	1 meal/month	1 meal/week (< 24")
	1 meal/month (≥ 24")	1 meal/month (> 24")		1 meal/month (> 24")
Lake trout	1 meal/month (< 30")	1 meal/week (≤ 22")	6 meals/year (< 24")	1 meal/month (< 30")
	Do not eat (≥ 30")	1 meal/month (22"-30")	Limited (> 24")*	Do not eat (> 30")
		Do not eat (> 30")		
Rainbow trout	1 meal/week (< 28")	1 meal/week	2 meals/month (< 20")	1 meal/week (< 28")
	1 meal/month (≥ 28")		6 meals/year (> 20")	1 meal/month (> 28")
Yellow perch	1 meal/week (< 11")	1 meal/week (≤ 11")	4 meals/month	1 meal/week (< 11")
	1 meal/week (≥ 11", GP)	1 meal/month (> 11")		1 meal/month (> 11")
	1 meal/month (≥ 11", SP)			

If not otherwise indicated, advisories are specific to Lake Michigan (rather than general statewide advisory). If both statewide and Lake Michigan advisories were available, the Lake Michigan advisory was recorded

*= Michigan employs a “limited” advisory category, which indicates that for the type of fish in question, SP should avoid consumption, and the GP should consume a maximum of one to two meals/year

**Table 3:** Fish consumption advisories for 11 common game fish in the Lower Mississippi River. If not otherwise indicated, advisories are specific to the Mississippi River (rather than general statewide advisory). If both statewide and Mississippi River advisories were available, the Mississippi River advisory was recorded.

	Arkansas	Tennessee	Missouri	Kentucky
Channel catfish	No advisory	Do not eat*	1 meal/week	1 meal/week (GP)^†^
				1 meal/month (SP)^†^
Flathead catfish	No advisory	Do not eat*	1 meal/week	1 meal/month (GP)^†^
				6 meals/year (SP)^†^
Blue catfish	No advisory	Do not eat*	1 meal/week (> 17")	1 meal/month (GP)^†^
				6 meals/year (SP)^†^
Common carp	No advisory	Do not eat*	1 meal/week (> 21")	1 meal/week (GP)^†^
				1 meal/month (SP)^†^
Black crappie	No advisory	Do not eat*	No Advisory	1 meal/week (GP)^†^
				1 meal/month (SP)^†^
White crappie	No advisory	Do not eat*	No Advisory	1 meal/week (GP)^†^
				1 meal/month (SP)^†^
Bluegill	No advisory	Do not eat*	No Advisory	1 meal/week (GP)^†^
				1 meal/month (SP)^†^
Freshwater drum	No advisory	Do not eat*	No Advisory	1 meal/week (GP)^†^
				1 meal/month (SP)^†^
Largemouth bass	No advisory	Do not eat*	1 meal/month (SP)^†^	1 meal/month (GP)^†^
				6 meals/year (SP)^†^
White bass	No advisory	Do not eat*	No Advisory	1 meal/month (GP)^†^
				6 meals/year (SP)^†^
Striped bass	No advisory	Do not eat*	No Advisory	1 meal/month (GP)^†^
				6 meals/year (SP)^†^

*= between Mississippi State Line and Meeman-Shelby State Park

† = from statewide advisory, not Mississippi River-specific

## Discussion

We compared risk assessment assumptions across US states to identify factors which lead to differences in advisories across jurisdictional boundaries. Our results show that differences in target tissue concentrations are influenced by three factors: (1) consideration of chronic non-cancer, chronic cancer, or acute health risk scenarios; (2) toxicity values (reference dose, cancer slope factor, acute tolerance level) which are applied in the risk assessment calculation; and (3) level of health risk a state tolerates. Collectively, these factors influence target tissue concentrations and discrepancies lead to conflicting advisory guidance, even within a single waterbody. Advisory discrepancies complicate public health messaging for how to safely consume self-caught fish and result in differential health protection. Our results are consistent with past comparative analysis of US advisory assumptions, which also have found substantial variation across states [29•, 46•, 47•, 48–49].

### Consideration of Different Risk Scenarios

At its core, risk assessment is a tool to evaluate health risk based on an exposure scenario — acute or chronic health and non-cancer or cancer health endpoints. While methylmercury advisories are limited to acute or chronic non-cancer health endpoints, PCB advisories may consider either acute, chronic non-cancer, or chronic cancer health risk, which leads to a wide range of difference across states. Sensitivity analyses demonstrate that advisories based on chronic cancer risk from PCBs generally result in more protective target tissue concentrations than those based on chronic non-cancer health endpoints for PCBs. However, recent evidence suggests the neurodevelopmental toxicity of PCBs [20] — a critical health consideration not currently reflected in the PCBs reference dose — may warrant re-evaluation of fish consumption advisories.

Once a health risk scenario has been selected, states calculate target tissue concentrations using Eqs. 1 or 2 to determine the amount of fish that people can safely consume from a region or waterbody. Our study identifies discrepancies in the numerical value of variables used by states when performing risk calculations by these equations. Of the four variables we considered, toxicity values (reference dose, cancer slope factor, acute tolerance level) were found to differ substantially across US states. Notably, methylmercury reference doses differ by as much as one order of magnitude. Sensitivity analysis shows that this class of input variables has the largest impact on target tissue concentrations. Toxicity values anchor a risk assessment calculation to epidemiologic and toxicological literature demonstrating adverse effects caused by exposure to the contaminant in question. States may either adopt a toxicity value from a cooperative or federal agency — such as the EPA, FDA, GLC, or ATSDR— or derive their own. For example, Alaska guidance assumes a methylmercury reference dose of 5.6 × 10^−4^ mg/kg-day, considering methylmercury to be five times less potent than the EPA-recommended reference dose of 1 × 10^−4^ mg/kg-day [74, 75]. The Alaska Scientific Advisory Committee for Fish Consumption derived this reference dose in 2014, when developing a unique risk assessment program to account for the cultural, economic, and nutritional reliance of native Alaskan populations on self-caught fish [75]. Given Alaska’s geographic separation from the continental USA, its population is subject to unique socioecological pressures, and a highly tailored fish consumption advisory program reflects an important effort to incorporate the best available science while also adequately protecting the intended population of fish consumers [38].

Advisories are also influenced by the level of health risk a state is willing to accept. For non-cancer health endpoints, all states in our analysis determine health risk to be unacceptable if an individual’s exposure to a contaminant via fish consumption is greater than the toxicity value (e.g., hazard quotient greater than 1). This implies that a consumer will only be exposed to a particular non-carcinogenic contaminant through fish consumption, which may not always be the case. For cancer health endpoints, states use different acceptable cancer risk levels, which is the most impactful variable in calculating carcinogenic risk from PCBs. A state that employs a cancer risk level of 10^−4^ accepts a 100-fold greater cancer risk than a state that chooses 10^−6^, resulting in substantially different target tissue concentrations and which diverge further from PCBs advisories based on non-cancer health endpoints. Bodyweight and meal size assumptions were also found to differ across the USA. However, it is not evident whether these assumptions are informed by state-level demographics or other factors, such as the policy-based level of health risk a state is willing to accept.

Differences in risk assessment assumptions across the three categories which we have described — health risk scenario, toxicity values, and health risk levels — have meaningful implications for the contaminant concentrations that trigger specific meal frequency advisories. Target tissue concentrations for methylmercury or PCB advisories of one meal/month or zero consumption differ by as much as two orders of magnitude across the USA. Because the EPA recommends that state agencies consider state-level data — including feasibility, efficacy, nutrition, cultural impacts, economic impacts, and adverse health outcomes — when developing an advisory program [76], it would be reasonable to see some variation in target tissue concentrations. Past analysis into the history of fish consumption advisories has argued that advisories in some states, like New York, were developed with recreational anglers in mind, rather than subsistence fishers [77]. New York uses national guidance when developing their advisories — including FDA acute tolerance levels, EPA toxicity values, and EPA meal size assumptions [78]. In contrast, guidance developed by the Alaska Scientific Advisory Committee for Fish Consumption directly addresses the dietary practices of native Alaskan populations [75]. Given the distinct motivations of each program, it is not surprising that New York and Alaska use different assumptions when developing fish consumption advisories. Further, Alaska and New York are over 4000 miles apart, and therefore any discrepancies in fish consumption advisory methods are unlikely to cause consumer confusion. State-level data — including known regional exposures, population demographics, and cultural dietary practices — may justifiably inform fish advisory assumptions, especially for states which are not in close geographic proximity.

### Inconsistent Public Health Messaging

Differences in risk assessment assumptions impact public health messaging about safe consumption of self-caught fish. The advisories published for a single waterbody bordered by contiguous states should be consistent in regions where those states are in close proximity, given the likely similarity in contaminant profiles and fish species. Our results show that this is not the case. Advisory discrepancies across states bordering Lake Michigan can in part be attributed to differences in advisory assumptions. While Illinois, Indiana, and Wisconsin use the GLC guidance document in establishing advisories, Michigan uses a state-specific guidance document [40, 41, 79]. Accordingly, Wisconsin, Illinois, and Indiana show relative consistency in their advisories, whereas Michigan diverges.

Differences in advisories across the four Lower Mississippi River states also reflect conflicting advisory assumptions. Arkansas utilizes a state-specific acute health-risk approach for methylmercury based on FDA standards, and a combination of guidance documents for PCBs including those released by ATSDR and EPA [42, 43, 80, 81]. Kentucky adopts EPA guidance for methylmercury and GLC guidance for PCBs [40, 82]. Missouri and Tennessee each use state-specific guidance for methylmercury and PCBs [72, 83]. In total, each state uses a different guidance document, including unique assumptions, to develop their advisories. The result is conflicting advisories for the Lower Mississippi River, where self-caught fish consumers may travel across state lines to fish and encounter conflicting guidance.

The Lower Mississippi River and Lake Michigan are just two examples of waterbodies bordered by multiple, contiguous states. The Ohio River experienced a similar challenge of different states issuing conflicting advisories for the same fishing areas and addressed this by establishing a uniform protocol [84]. The GLC technical guidance document endeavors to serve an analogous function, but some Great Lakes states (Michigan, New York, and Pennsylvania) use state-specific or other guidance [40, 41, 78, 79, 85], which creates an opportunity for discrepancies and confusion among self-caught fish consumers in the Great Lakes region. We recognize that state environmental protection and public health agencies work hard to protect the health of their populations and are limited by factors beyond their control. However, to alleviate this confusion, it is important that states in regions such as the Lower Mississippi River and the Great Lakes continue to work to identify opportunities for inter-state consistency and collaboration.

Our comparative analysis is meaningful because it aggregates information about advisories across 46 of 50 states, representing 92 percent of the USA. We highlight how differences in advisories manifest even within single waterbodies bordered by contiguous states with or without regional advisory guidance. Our scope was limited to the information contained within technical fish consumption advisory guidance documents, which is anticipated to include relevant information for creating advisories. However, some states may embed additional details in other documents or resources, and these nuances are not captured within this study. In addition, our work does not consider the communication tools and techniques used to disseminate advisories. Since some dissemination methods are known to be more effective than others at informing people of health risks posed by self-caught fish [86, 87], future research should consider risk communication. Our analysis focused on four key risk assessment variables shown in Eqs. 1 and 2, but did not capture additional variables that some states may use in calculating advisories. Some advisory discrepancies may be influenced by these additional variables, such as time, cooking reduction, and absorption factors. Finally, our work does not consider advisory implementation challenges, such as differences in the quality of fish tissue contaminant data due to sampling frequency, spatial coverage, measured analytes, and local sampling resources, which we recognize are potentially important sources of difference in advisories issued by contiguous states for the same body of water. We encourage future researchers to investigate fish tissue monitoring programs across US states to understand the influence of fish tissue contaminant data on fish consumption advisories. We also recommend that future research evaluate the basis for each risk assessment variable used in current fish consumption advisory guidance documents to reflect current recommendations for how to incorporate best available science into the risk assessment process [38].

## Conclusion

Our review and analysis of advisories across US states identifies the major risk assessment factors which contribute to disparities in advice for consumers of self-caught fish. Our work shows that US state fish consumption advisory programs differ in their fundamental interpretation of the science of toxicity. We also highlight and recommend the utility of regional collaborations when deriving advisories in order to provide consistent public health messaging to consumers. This is especially important for contiguous states that border the same waterbody, and for messaging directed toward SP of fish consumers. We hope that this work will inspire neighboring states to collaborate in setting advisories so that consumers receive consistent messaging regarding their fish intake. Ultimately, clear and concise public health messaging is key to helping consumers eat self-caught fish in a manner that promotes the health benefits of doing so, while minimizing health risks associated with exposure to contaminants found in fish tissue.

## Supplementary Information

ESM 1 (PDF 113 kb)

## Data Availability

All data presented herein was obtained from publicly available documents. Investigators interested in these documents are referred to the respective state, federal, and consortium websites. For other questions, investigators should contact Kathryn Crawford (kcrawford@middlebury.edu).
