# Supplementary material for: Comparison of Recreational Fish Consumption Advisories Across the USA
Source: Curr Environ Health Rep. 2021 May 1;8(2):71–88. doi: 10.1007/s40572-021-00312-w (PMC8208921; doi:10.1007/s40572-021-00312-w)

Supplemental Material

Comparison of recreational fish consumption advisories across the United States

Brittany M. Cleary, Megan E. Romano, Celia Y. Chen, Wendy Heiger-Bernays, Kathryn A. Crawford

Table of Contents

Page 2. Table S1: Summary of target tissue concentration obtainment method

Page 3. Figure S1a: Distribution of publication year of methylmercury technical guidance documents
Figure S1b: Distribution of publication year of PCBs technical guidance documents

Page 4. Figure S2a: Distribution of methylmercury health risk scenarios

Page 5. Figure S2b: Distribution of PCBs health risk scenarios

Page 6. Figure S3a: Distribution of methylmercury reference dose assumptions
Figure S3b: Distribution of PCBs reference dose assumptions

Page 7. Figure S4a: Distribution of PCBs cancer slope factor assumptions
Figure S4b: Distribution of PCBs cancer risk level assumptions

Page 8. Figure S5a: Distribution of GP bodyweight assumptions
Figure S5b: Distribution of WCBA bodyweight assumptions

Page 9. Figure S5c: Distribution of young children bodyweight assumptions

Page 10. Figure S6a: Distribution of GP/WCBA meal size assumptions
Figure S6b: Distribution of young children meal size assumptions

Page 11. Figure S7a: Sensitivity analysis for methylmercury chronic noncancer health risk scenario

Page 12. Figure S7b: Sensitivity analysis for PCBs chronic noncancer health risk scenario
Figure S7c: Sensitivity analysis for PCBs chronic cancer health risk scenario

**Key**	
BW	Bodyweight
GP	General population
SP	Sensitive populations
WCBA	Women of childbearing age
MS	Meal size
RfD	Reference dose
ATL	Acute tolerance level
CSF	Cancer slope factor
RL	Cancer risk level

Table S1. Summary of target tissue concentration obtainment method.

**Target Tissue Concentration**	**Total [a] – n states**	**Abstracted [b] – n states (% of total)**	**Calculated [c] – n states (% of total)**
Methylmercury			
Zero consumption	41	31 (76)	10 (24)
GP	41	31 (76)	10 (24)
WCBA	7	3 (43)	4 (57)
Young Children	8	3 (38)	5 (62)
Overall SP	12	10 (83)	2 (17)
One meal/month	35	25 (71)	10 (29)
GP	35	25 (71)	10 (29)
WCBA	7	3 (43)	4 (57)
Young Children	8	3 (38)	5 (62)
Overall SP	8	6 (75)	2 (25)
PCBs			
Zero consumption	34	27 (80)	7 (20)
GP	34	27 (80)	7 (20)
WCBA	4	1 (25)	3 (75)
Young Children	5	1 (20)	4 (80)
Overall SP	3	3 (100)	0 (0)
One meal/month	29	22 (77)	7 (26)
GP	29	22 (77)	7 (26)
WCBA	4	1 (25)	3 (75)
Young Children	5	1 (20)	4 (80)
Overall SP	3	3 (100)	0 (0)

[a] Total = number of states presented in **Figures 1a-b and 2a-b**
[b] Abstracted = number of states which reported target tissue concentrations in their technical guidance document(s)
[c] Calculated = number of states for which we calculated target tissue concentrations using information contained within respective technical guidance documents.

Figure S1(a-b). Distribution of publication year for **(a)** methylmercury technical guidance documents (n = 42 states) and **(b)** PCBs technical guidance documents (n = 40 states).

Figure S1a.

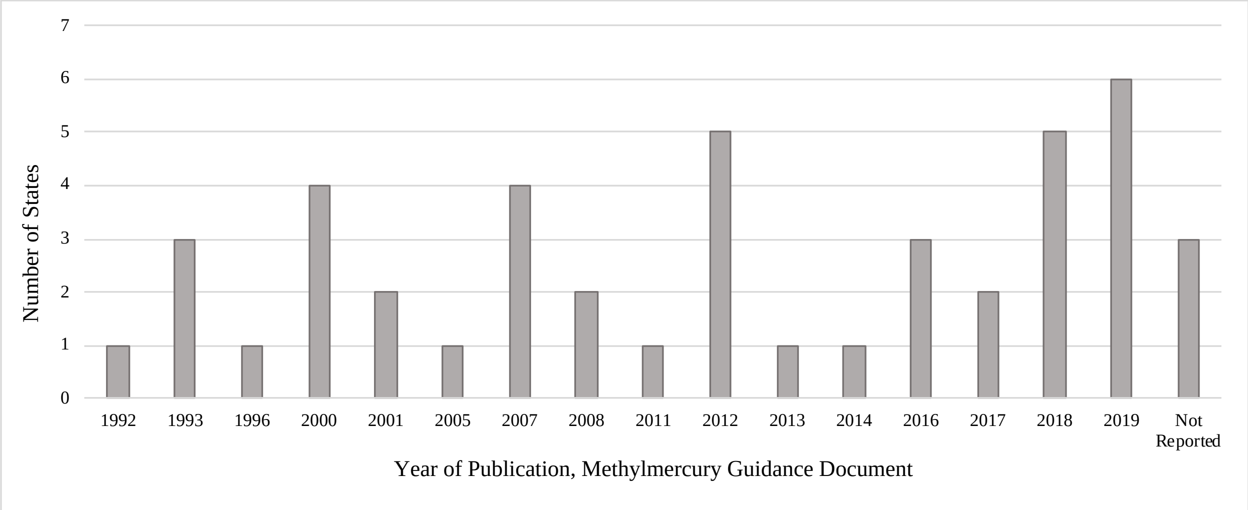

Figure S1b.

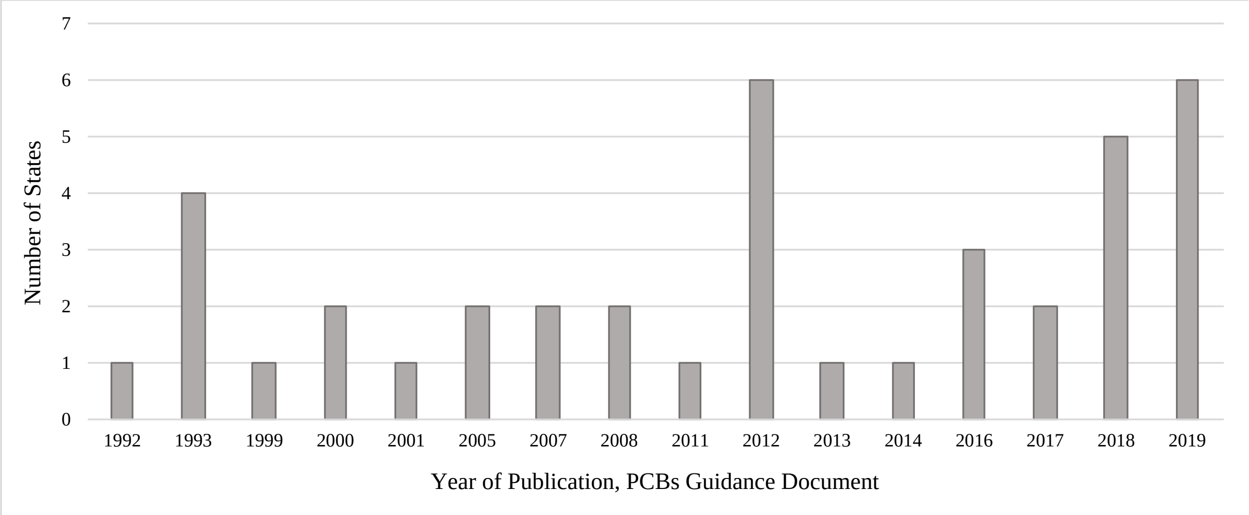

Figure S2(a-b). Distribution of health risk scenario for development of **(a)** methylmercury (n = 46 states) and **(b)** PCB (n = 46 states) fish consumption advisories. Unspecified = state considers the specific contaminant when developing fish consumption advisories, but did not report information about their health risk scenario. No Advisory = state does not consider a specific contaminant when developing fish consumption advisories

Figure S2a.

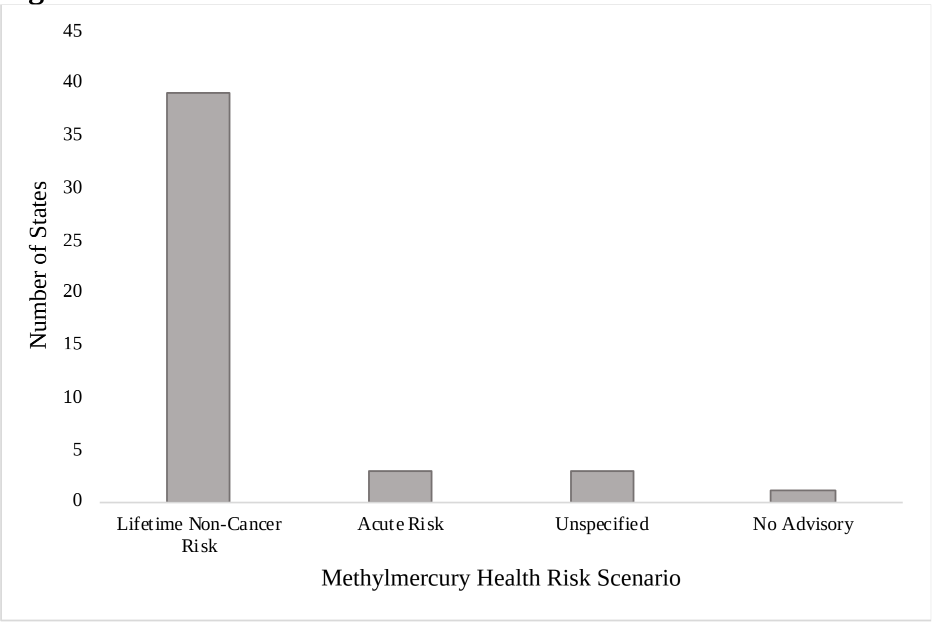

Figure S2b.

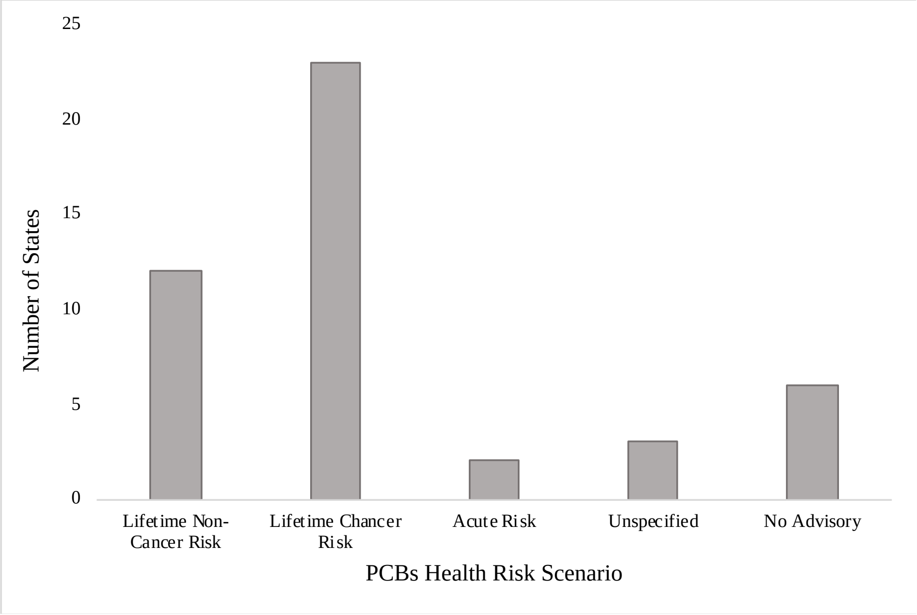

25
20
15
10
5
0
Number of States
Lifetime Non-Cancer Risk
Lifetime Chancer Risk
Acute Risk
Unspecified
No Advisory
PCBs Health Risk Scenario

Figure S3(a-b). Distribution of reference dose for states that consider non-cancer risk of **(a)** methylmercury (n = 39 states) and **(b)** PCBs (n = 13 states).

Figure S3a.

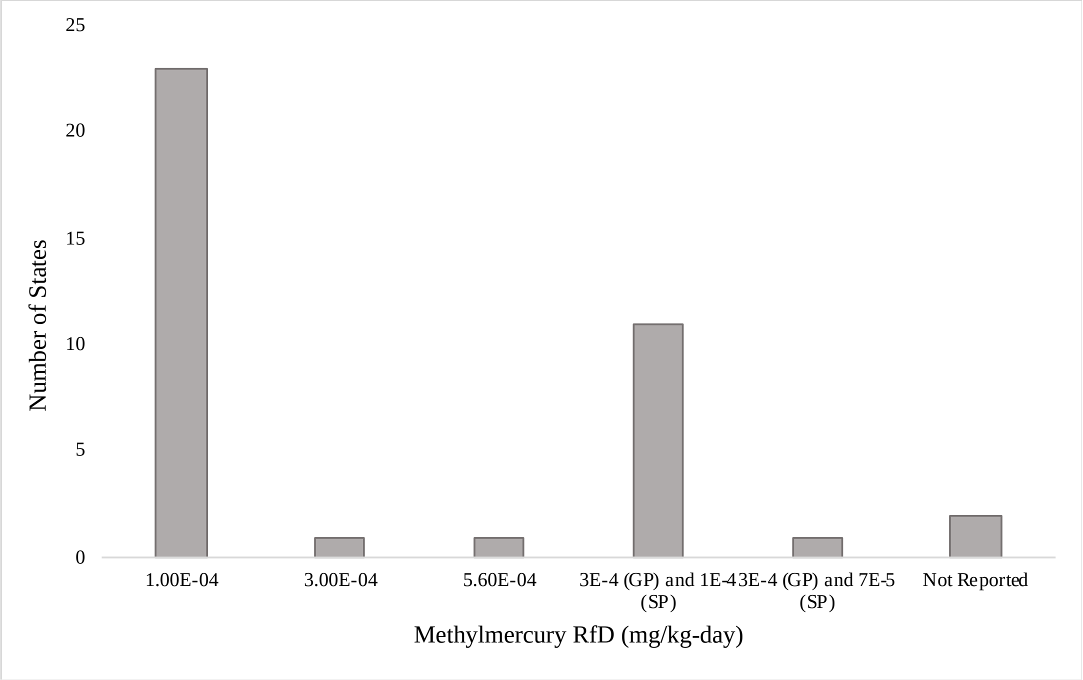

Figure S3b.

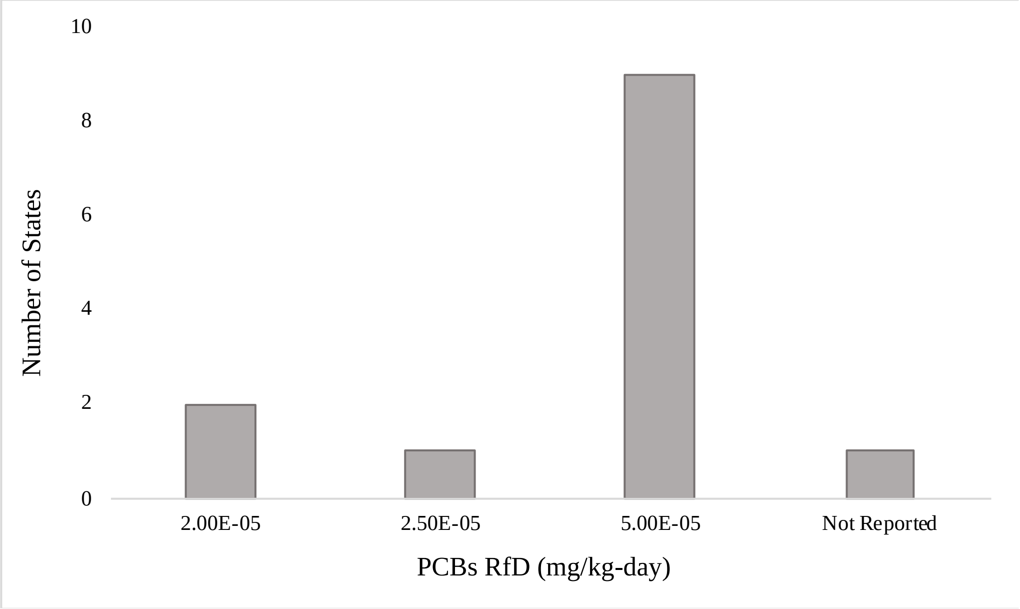

Figure S4(a-b). Distribution of **(a)** PCB cancer slope factor and **(b)** cancer risk level across n = 23 states which consider chronic cancer risk of PCBs.

Figure S4a.

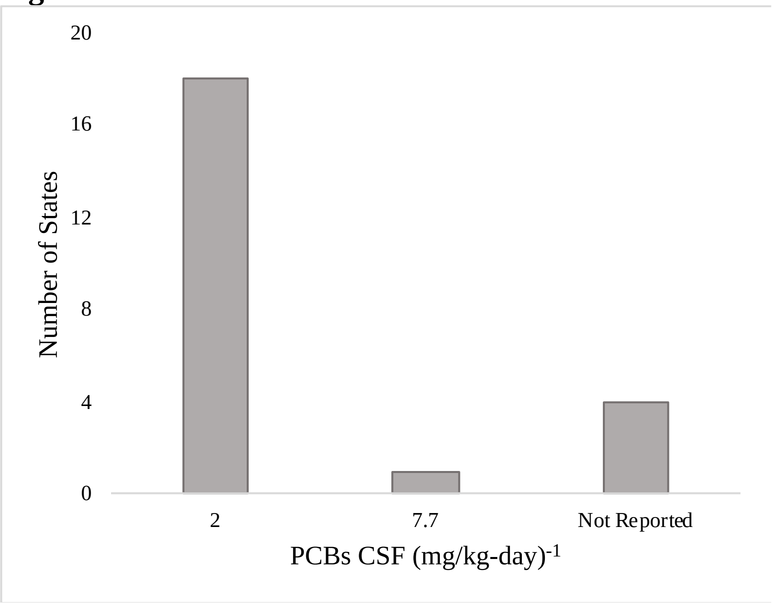

Figure S4b.

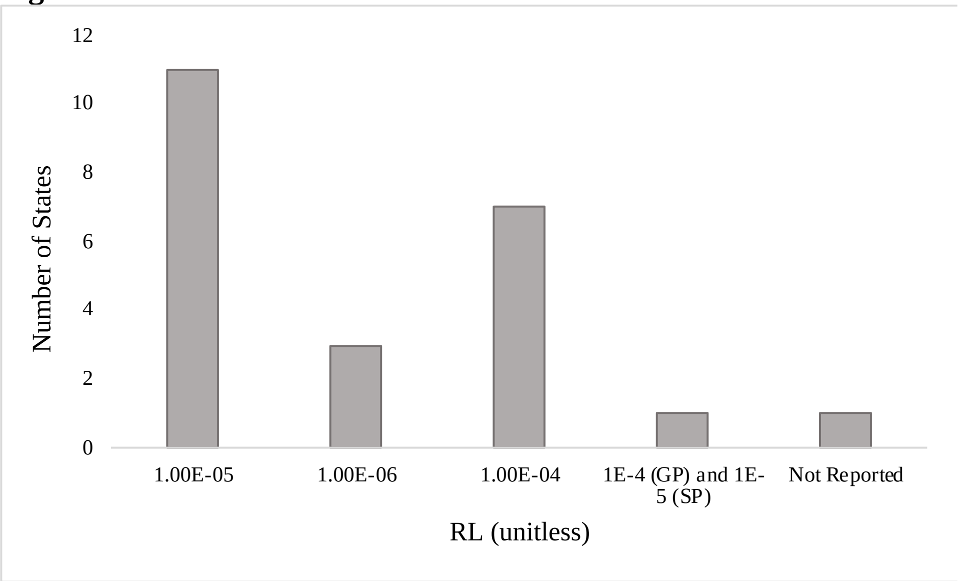

Figures S5(a-c). Distribution of bodyweight assumptions for **(a)** GP (n = 39 states), **(b)** WCBA (n = 39 states), and **(c)** young children (n = 39 states).

Figure S5a.

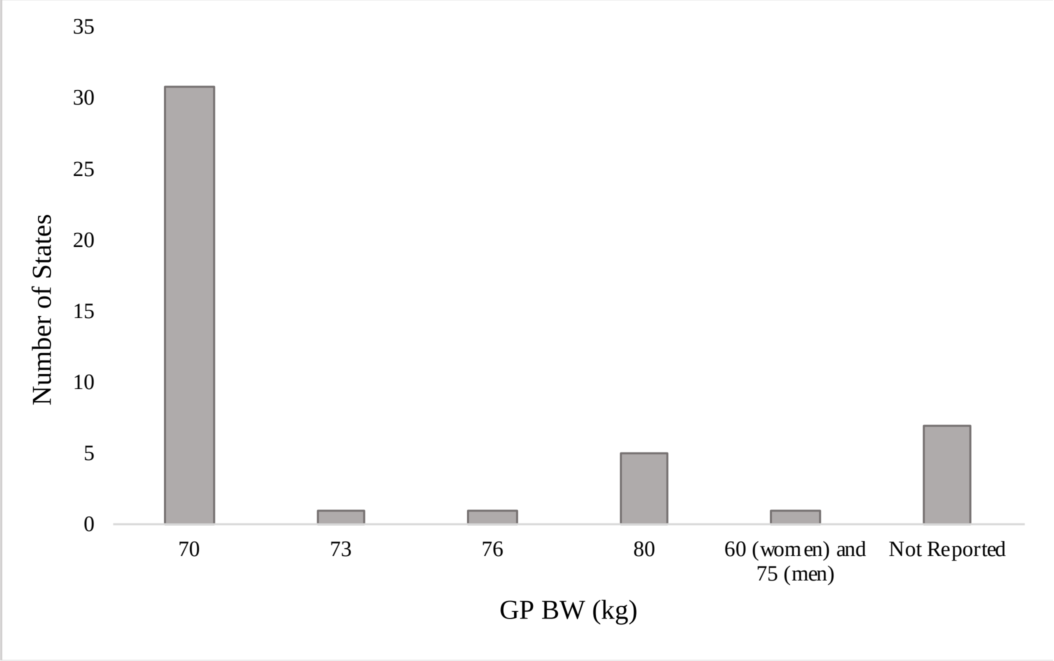

Figure S5b.

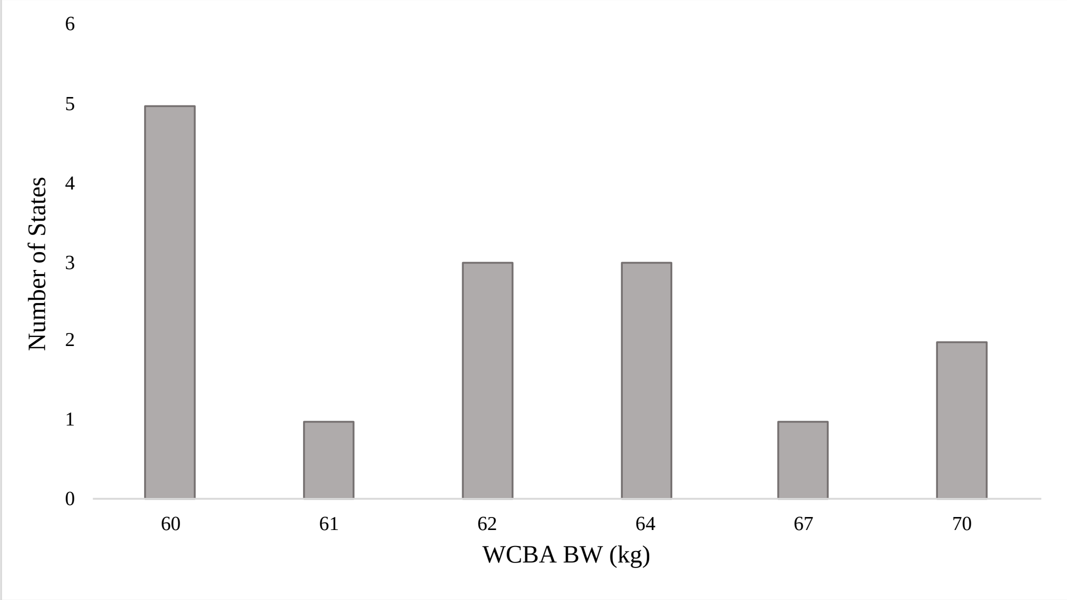

Figure S5c.

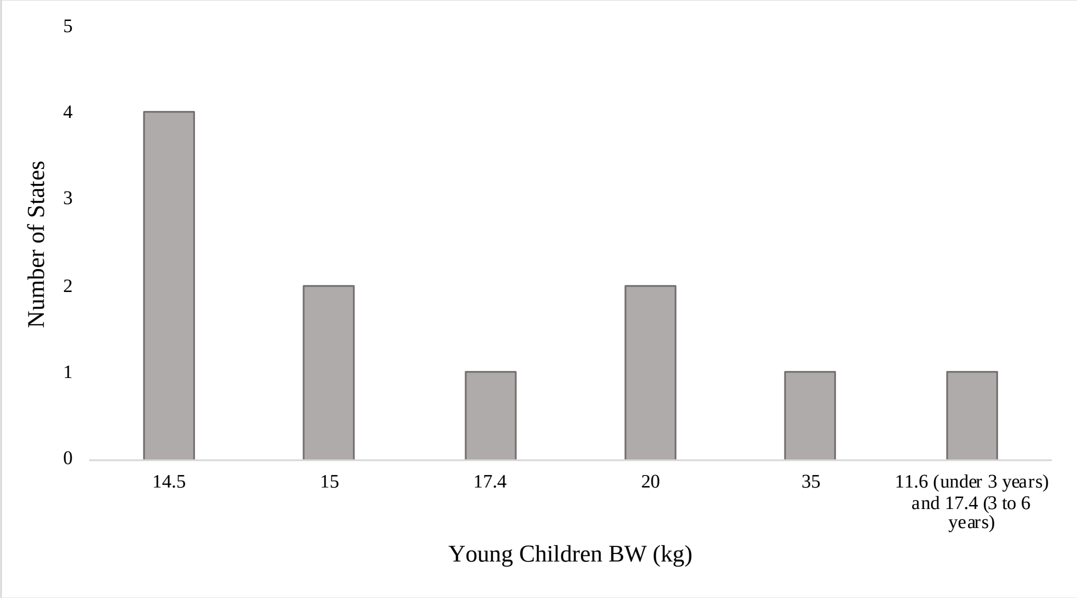

5
4
3
2
1
0
Number of States
14.5
15
17.4
20
35
11.6 (under 3 years) and 17.4 (3 to 6 years)
Young Children BW (kg)

Figures S6(a-b). Distribution of meal size assumptions for **(a)** GP and WCBA (n = 39 states) and **(b)** young children (n = 9 states).

Figure S6a.

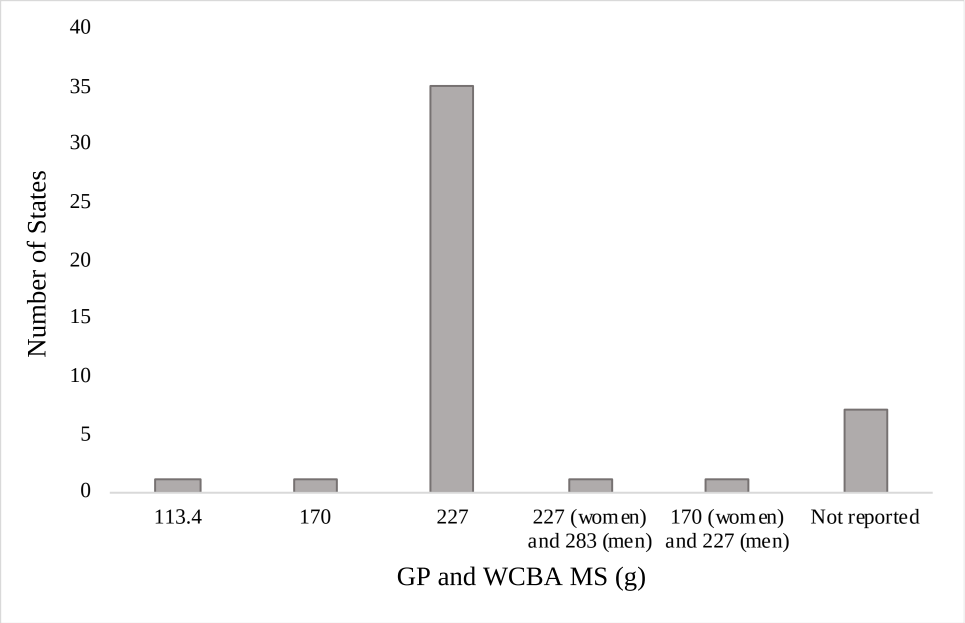

Figure S6b.

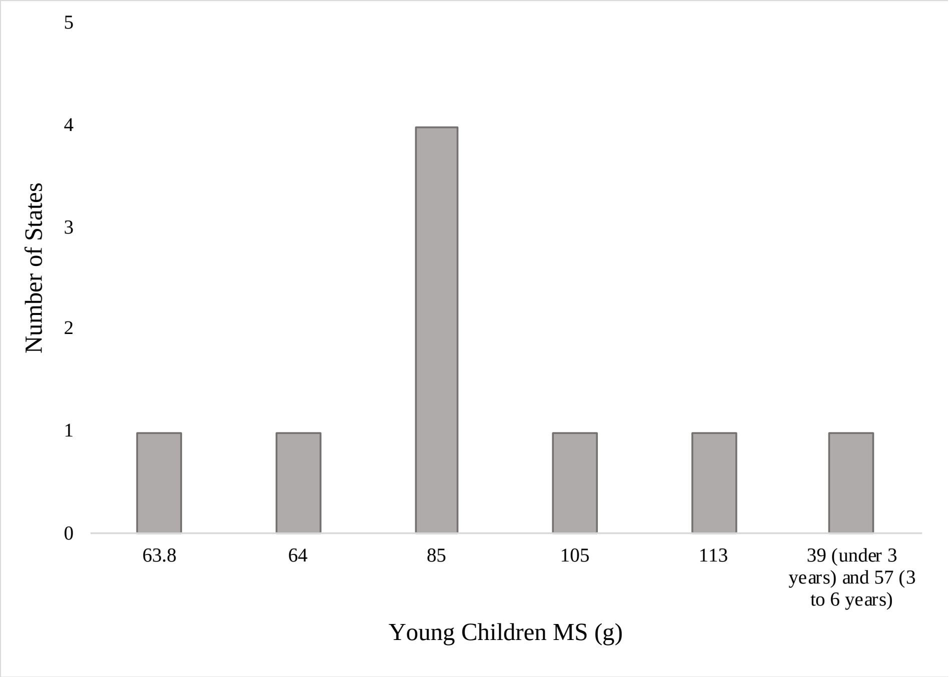

Figures S7(a-c). Sensitivity analysis of risk assessment parameters for **(a)** methylmercury chronic non-cancer health endpoints, **(b)** PCBs chronic non-cancer health endpoints, and **(c)** PCBs chronic cancer health endpoints. Bars represent the difference between the least and most protective theoretical target tissue concentration (for a one meal/month advisory) based on the maximum and minimum values of one parameter, holding all other parameters at their median value.

Figure S7a.

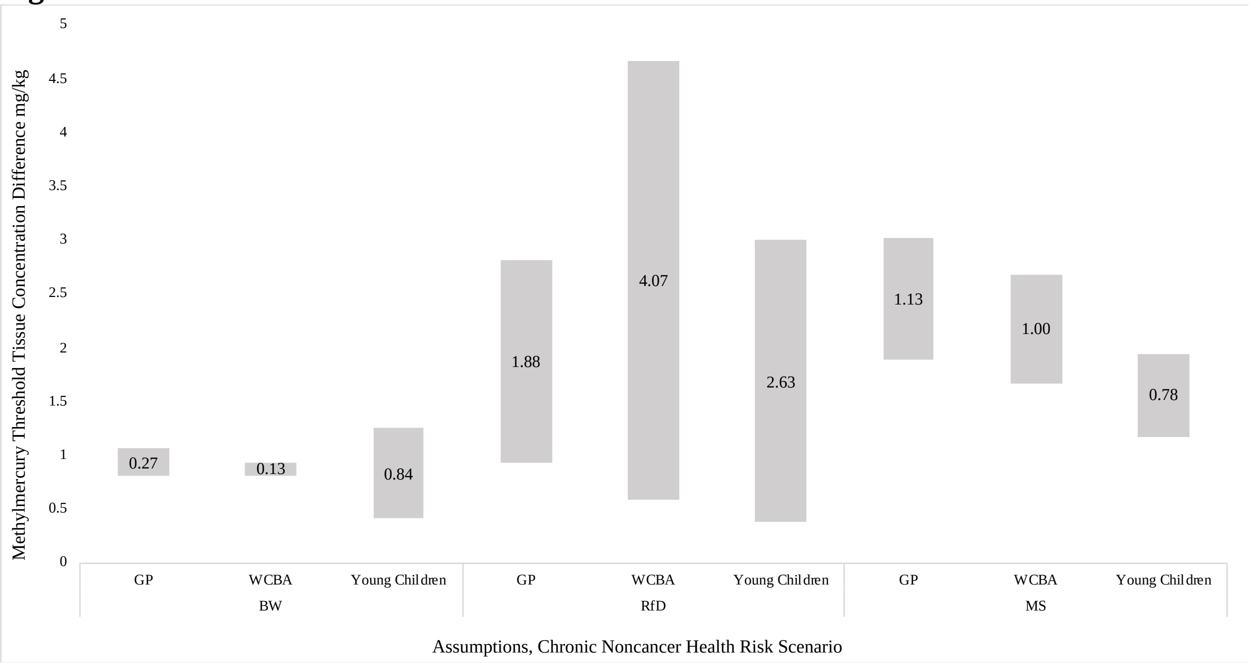

Figure S7b.

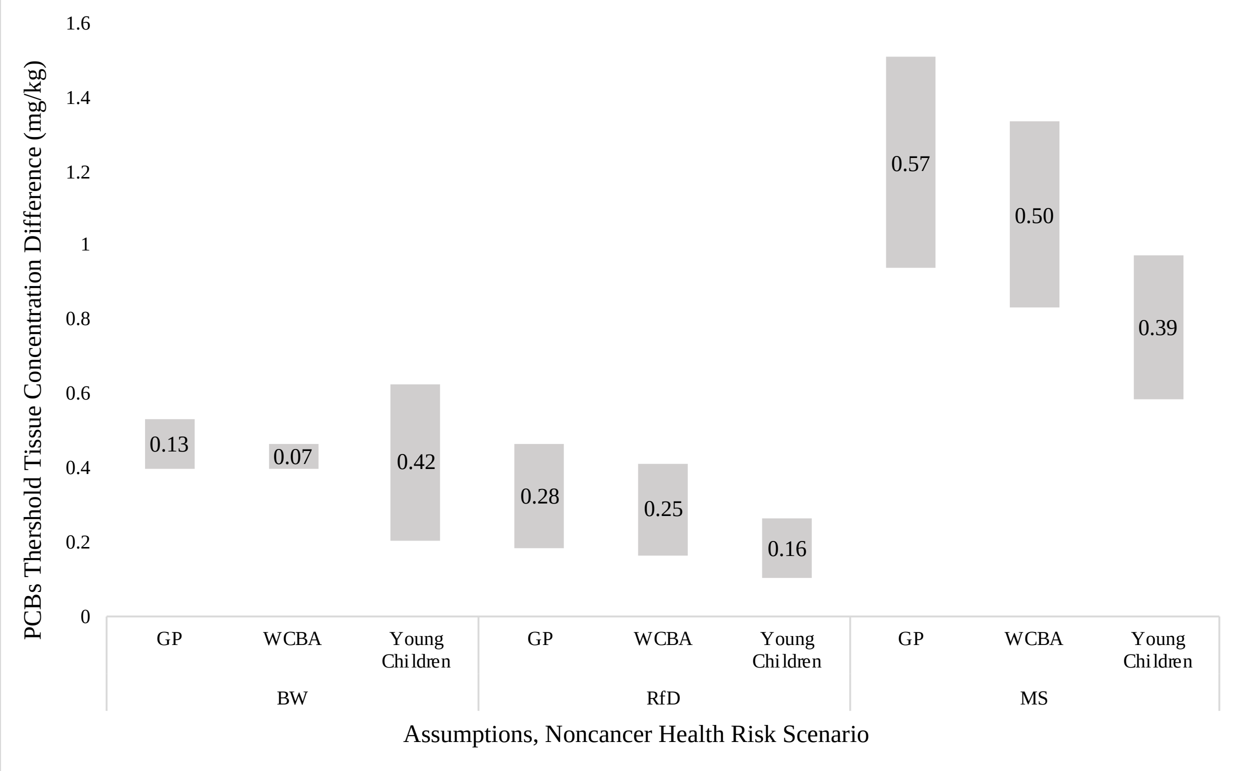

Figure S7c.

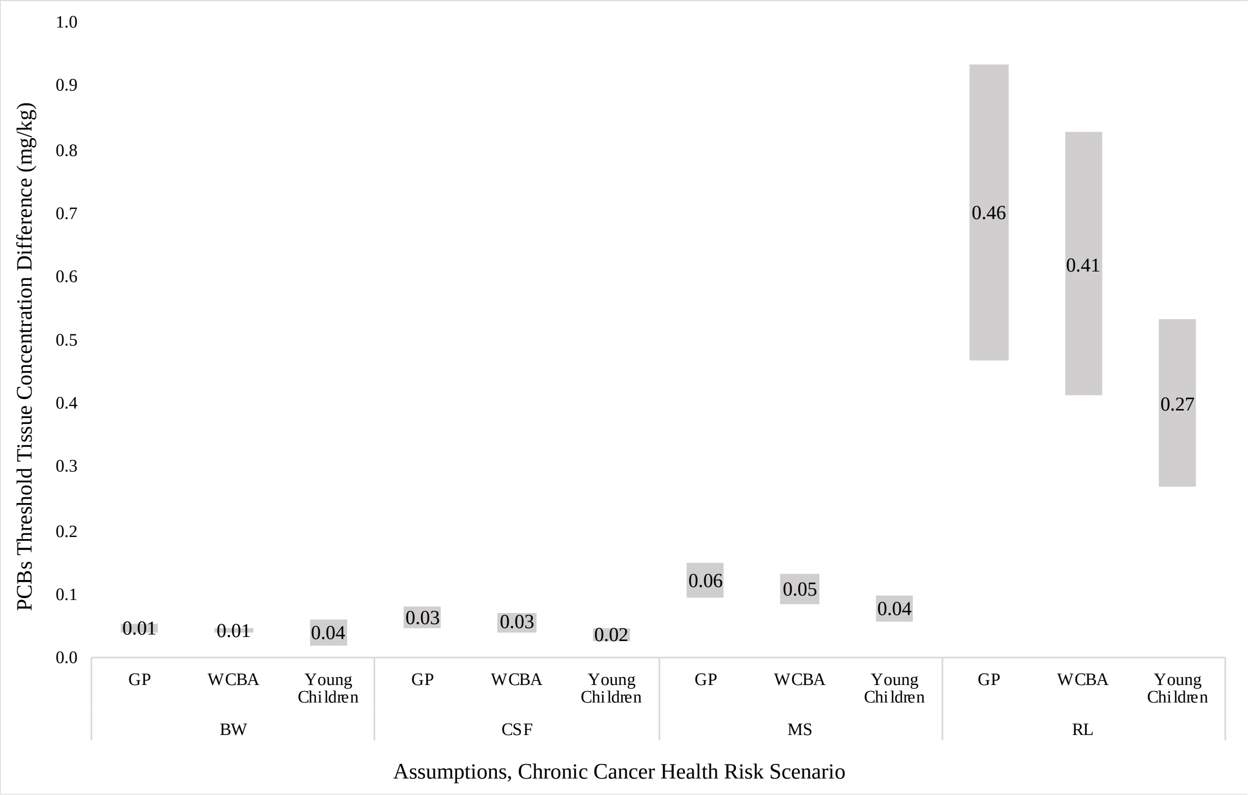

Supplement: Supplementary file 1 — (PDF 113 kb) [file 40572_2021_312_MOESM1_ESM.pdf]
